# LAP-IoHT: A Lightweight Authentication Protocol for the Internet of Health Things

**DOI:** 10.3390/s22145401

**Published:** 2022-07-20

**Authors:** Chien-Ming Chen, Zhaoting Chen, Saru Kumari, Meng-Chang Lin

**Affiliations:** 1 College of Computer Science and Engineering, Shandong University of Science and Technology, Qingdao 266590, China; chienmingchen@ieee.org (C.-M.C.); SDKJDXchen@163.com (Z.C.); 2 Department of Mathematics, Chaudhary Charan Singh University, Meerut 250004, Uttar Pradesh, India; saryusiirohi@gmail.com; 3 Graduate Institute of Nanomedicine and Medical Engineering, College of Biomedical Engineering, Taipei Medical University, Taipei 115, Taiwan

**Keywords:** Internet of Health Things, authentication, network security

## Abstract

The Internet of Health Things (IoHT), which is an extension of the Internet of Things (IoT) in healthcare, has provided a new type of telemedicine approach. In IoHT, wearable sensors are used to collect patient health data, and information is transmitted remotely to doctors who can develop accurate treatment plans and provide timely telemedicine services to patients. However, patient health data are transmitted over a public channel, which means that the privacy and medical data of patients are at significant risk of leakage and can be confronted by serious security problems. We proposed a lightweight authentication protocol known as LAP-IoHT for IoHT environments to overcome the various threats that are currently faced by IoHT. We verified the security of LAP-IoHT using a Real-or-Random model and demonstrated its significant performance advantage by conducting a comparative analysis with other similar protocols for a better adaptation to the IoHT environment.

## 1. Introduction

The rapid development of communication technologies has resulted in the extensive application of the Internet of Things (IoT) [1,2,3,4]. By using wireless networks to connect devices and various servers, IoT [5] provides a new means of communication that further enables interaction between virtual environments and the real world. Sensors [6,7] are the most common and versatile IoT devices. Wireless sensor networks (WSNs) [8,9,10] consist of numerous sensors to monitor specific areas and collect data. Hence, sensors and WSNs play an essential role in IoT development. At present, IoT is widely deployed in various applications and environments, such as manufacturing [11], environmental protection [12], smart cities [13,14], and intelligent transportation [15,16]. The rapid increase in the number of IoT devices demonstrates the importance and development potential of IoT, which is gradually improving the quality of life and making intelligent living and digital life possible.

Furthermore, the Internet of Health Things (IoHT) [17,18], which is a subset of IoT, is used extensively in healthcare scenarios [19,20,21]. In IoHT, wearable sensors [22,23] are implanted into the human body or set on body surfaces depending on the disease condition, thereby continuously monitoring the physiological indicators of the patient. These wearable sensors collect real-time data from the human body and transmit them to servers. Doctors can remotely analyze these data in order to provide timely medical services to patients. As the development of the healthcare sector is closely linked to people’s lives, IoHT can prevent several chronic diseases, save patient transportation costs, protect the health of healthcare professionals, reduce the possibility of conflicts between doctors and patients, and help family members to remain abreast of patients’ current conditions. IoHT provides higher-quality healthcare services, improves the level and efficiency of services, and optimizes the use of healthcare resources.

Security and privacy [24,25,26,27] have become the primary challenges of IoHT. In an IoHT system, the medical information of patients collected by sensors is transmitted over open networks. Since this information is highly sensitive, it must be protected from unauthorized users or malicious attackers, who may steal, modify, and delete health data, corrupt medical records, and even threaten the lives of patients. Moreover, attackers may target medical devices by hijacking and forging such devices, resulting in the denial of service and, in severe cases, possible damage to medical devices. Therefore, exploring a security mechanism to address the current environment and eliminate threats in IoHT is necessary.

This study proposed a lightweight authentication protocol (LAP) known as LAP-IoHT for IoHT environments. In LAP-IoHT, all participants, including the users and wearable sensors, are authenticated by the gateway. Subsequently, a shared session key is established for each communication session. LAP-IoHT encrypts the biometric features of the users to ensure anonymity. To demonstrate the security and reliability of this approach, we applied the Real-or-Random (ROR) model to analyze LAP-IoHT. The experimental results indicated that LAP-IoHT exhibits improved communication and computationally efficient performance.

The main contributions of this study are as follows:

(1) To address the current security issues frequently encountered in healthcare IoT systems, we designed a three-factor IoHT-based protocol that incorporates authentication and key negotiation, thereby guaranteeing privacy and access control.
(2) The introduction of biometrics, which protects the anonymity of users with unique information, can provide better user experience and privacy protection. In addition to using common one-way hash functions and simple XOR operations, we adopted asymmetric encryption and decryption in the protocol to provide higher security.
(3) Based on a shared ROR model, we performed a formal security analysis to evaluate the security, soundness, and integrity of the session key and protocol. Moreover, the informal security analysis provided strong evidence that the protocol is resistant to currently known security attacks.
(4) We conducted a comparative study and analyzed the performance of several protocols of the same type, taking into account the computational cost, time efficiency, and security properties. The results demonstrated that our protocol exhibits a significant performance advantage.

The remainder of this paper is organized as follows: Section 2 describes related work. In Section 3, we outlined the proposed LAP-IoHT protocol. Section 4 and Section 5 provide the security analysis and performance evaluation, respectively. Finally, Section 6 concludes the paper.

## 2. Related Work

IoT is widely adopted in healthcare monitoring systems. Onasanya et al. [28] proposed an IoT healthcare system for cancer care. Sun et al. [29] developed a medical record search protocol for IoT healthcare to ensure privacy preservation. Zhang et al. [30] proposed an isolation computing technology for cloud-based IoT healthcare. In 2020, Selvaraj et al. [31] reviewed the challenges and opportunities in IoT healthcare systems. Furthermore, several researchers have emphasized security and privacy issues. In 2019, Alassaf et al. [32] simulated the implementation of cryptographic functions for data in IoT healthcare. Kumari et al. [33] described a secure framework for medical systems in 2020. In 2021, Hossien et al. [34] introduced a privacy-preserving architecture for IoT healthcare based on blockchain. Wang et al. [35] proposed privacy preservation in IoT-enabled healthcare systems.

Moreover, several authentication protocols are available for IoHT. A summary of the applications of IoT in the medical industry is presented in Table 1. In 2015, Amin et al. [36] argued that elliptic curve cryptography could provide improved security for IoHT, but the protocol was not resistant against offline password-guessing attacks and privileged insider attacks. Challa et al. [37] proposed a three-factor authentication protocol for IoHT in 2018. However, once the sensor node was obtained by a malicious attacker, it broke the security of the protocol [37]. In 2019, Preeti et al. [38] designed a protocol that applied a WSN to IoHT and used a smart card. However, their protocol did not provide perfect forward security or resistance against sensor node capture attacks. Aghili et al. [39] proposed an access control and ownership transfer protocol for IoHT systems. Unfortunately, Amintoosi et al. [40] pointed out that the protocol of Aghili et al. [39] could not provide perfect forward security and was vulnerable to malicious sensor and server spoofing attacks. They also proposed a low-cost protocol for IoHT. In 2019, Gupta et al. [41] proposed a protocol that used wearable medical devices for IoHT to prevent attackers from modifying patient health information. However, Hajian et al. [42] pointed out that this protocol [41] did not protect information against privileged insider attacks, offline password-guessing attacks, and de-synchronization attacks. The proposed protocol of Hajian et al. [42] also could not provide perfect forward security and was vulnerable to session-key disclosure and impersonation attacks. To improve the security of the protocol, Kumar et al. [43] used digital signatures to encrypt the IoHT protocol communication process. Recently, Yu et al. [44] proposed a more realistic application-compliant authentication protocol designed around blockchain and physically unclonable functions while also enhancing mutual authentication between entities.

## 3. Proposed LAP-IoHT

### 3.1. Network Model

**Figure 1:**
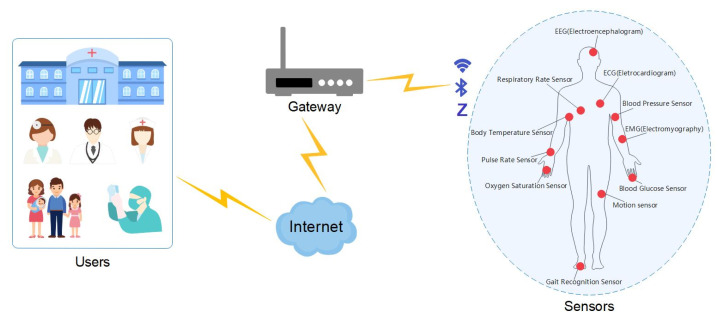
System model.

Figure 1 depicts the overall network model of the proposed protocol. This model describes a typical IoHT environment. The architecture includes three entities: users, a gateway, and wearable sensors:

(1) Wearable sensors are set on the bodies of patients. They can observe various body indicators, such as the electrocardiogram (ECG), electromyography (EMG), electroencephalogram (EEG), respiratory rate, pulse, blood pressure, blood glucose, and oxygen saturation. These wearable sensors should be registered with a gateway before being deployed to human bodies for precise management.
(2) Users are organizations or groups of people who can view the health data of patients. For example, users may be hospital administrators, doctors, pharmacists, nurses, families of patients, data analysts, and drug trialists. If a person needs to enter the network and view patient medical data, the person must register with the gateway in advance and become a legitimate user with the appropriate authorities.
(3) The gateway in our IoHT architecture acts as a trusted server. Prior to entering this network, all wearable sensors and users should register with the gateway. Subsequently, the gateway manages the list of all sensors and legitimate users.

Assume that a user desires to obtain data from a specific wearable sensor. This user transmits a request to the gateway and the gateway forwards this request to the sensor. After receiving the request, the wearable sensor sends the data to the user with the help of the gateway. Since medical data are personal and private, all communications among the users, gateway, and sensors should be confidential. The most straightforward method for achieving this is to encrypt the transmitted data.

The gateway can authenticate users and sensors using the proposed protocol. Moreover, a shared session key is established for each session.

### 3.2. LAP-IoHT

This section presents the proposed LAP-IoHT protocol for IoHT, which consists of three phases: user registration, sensor registration, and login and authentication. The notations and symbols are defined in Table 2.

### 3.3. User Registration Phase

**Figure 2:**
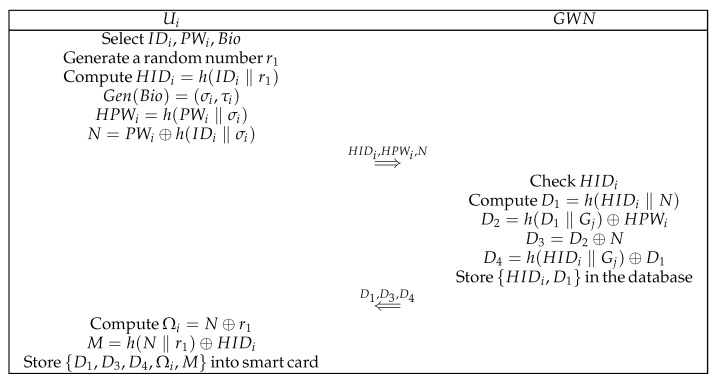
User registration phase.

Assume that user $U_{i}$ desires to become a legitimate user. This user must register with GWN. Figure 2 shows the steps that are involved in this phase. The messages are transmitted through a secure channel.

(1) $U_{i}$ prepares his or her own $ID_{i}$ and $PW_{i}$ and unique biometric Bio and selects a random number $r_{1}$. Subsequently, $U_{i}$ computes $HID_{i}=h\left(ID_{i}\|r_{1}\right)$, $Gen\left(Bio\right)=\left(\sigma_{i},\tau_{i}\right)$, $HPW_{i}=h\left(PW_{i}\|\sigma_{i}\right)$, and $N=PW_{i}⊕h\left(ID_{i}\|\sigma_{i}\right)$. Thereafter, $U_{i}$ transmits $\{HID_{i}$, $HPW_{i}$, N} to GWN.
(2) GWN first verifies whether $HID_{i}$ has already been registered. Thereafter, GWN calculates $D_{1}=h\left(HID_{i}\|N\right)$, $D_{2}=h\left(D_{1}\|G_{j}\right)⊕HPW_{i}$, $D_{3}=D_{2}⊕N$, and $D_{4}=h\left(HID_{i}\|G_{j}\right)⊕D_{1}$. Subsequently, GWN stores $\left\{HID_{i},D_{1}\right\}$ in its database and transmits $\{D_{1}$, $D_{3}$, $D_{4}\}$ to $U_{i}$.
(3) $U_{i}$ computes $\Omega_{i}=N⊕r_{1}$ and $M=h\left(N\|r_{1}\right)⊕HID_{i}$, and then stores $\{D_{1}$, $D_{3}$, $D_{4}$, $\Omega_{i}$, M} in his or her smart card.

### 3.4. Sensor Registration Phase

**Figure 3:**
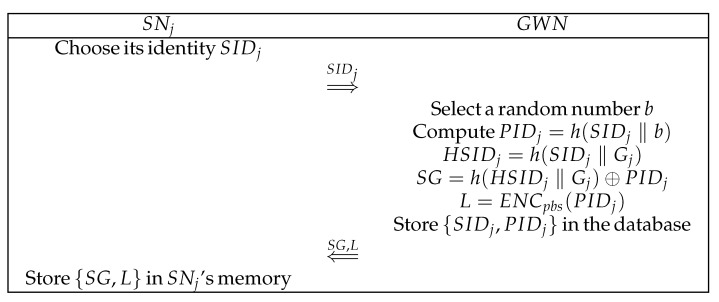
Sensor registration phase.

A wearable sensor must also be registered before joining the network. Assume that sensor $SN_{j}$ desires registration with GWN. Figure 3 depicts the detailed steps involved in this phase. The messages are submitted via a secure channel:

(1) $SN_{j}$ sends its identity $SID_{j}$ to GWN.
(2) GWN generates a random number *b* and calculates the pseudo-identity $PID_{j}$ of $SN_{j}$, where $PID_{j}=h\left(SID_{j}\|b\right)$. Subsequently, GWN calculates $HSID_{j}=h\left(SID_{j}\|G_{j}\right)$ and $SG=h\left(HSID_{j}\|G_{j}\right)⊕PID_{j}$ with its own private key $G_{j}$. GWN also uses an asymmetric encryption system to encrypt PID with the public key of $SN_{j}$. At this point, GWN calculates $L=ENC_{pbs}\left(PID_{j}\right)$, sends {SG, L} to $SN_{j}$, and stores $\{SID_{j}$, $PID_{j}\}$ in the database.
(3) $SN_{j}$ stores {SG, L} in its own memory.

### 3.5. Login and Authentication Phase

**Figure 4:**
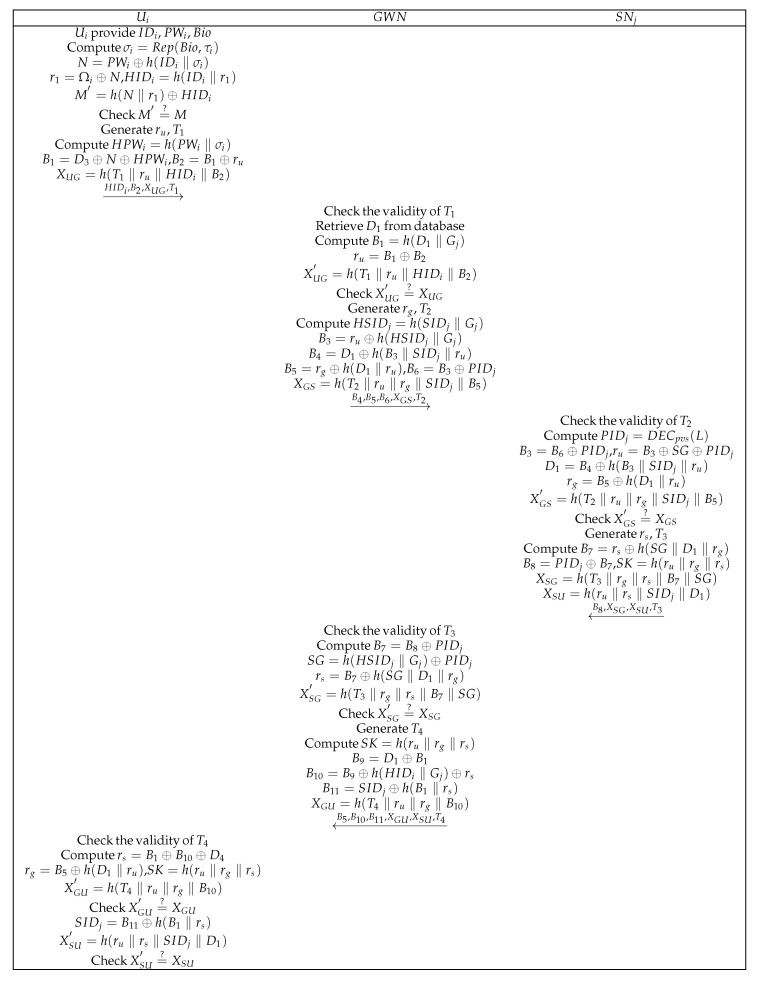
Login and authentication phase.

If $U_{i}$ requires connection to a specific wearable sensor $SN_{j}$, GWN needs to verify the legitimacy of the user. Subsequently, $U_{i}$, GWN, and $SN_{j}$ build a session key to encrypt the messages among them. In this phase, several parameters (e.g., $M^{{}^{\prime }}$, $X_{UG}^{{}^{\prime }}$, $X_{GS}^{{}^{\prime }}$, $X_{SG}^{{}^{\prime }}$, and $X_{Gu}^{{}^{\prime }}$) are calculated. Figure 4 illustrates this phase, the details of which are as follows:

(1) $U_{i}$ inserts his or her smart card into a smart card reader/computer and provides his or her identity $ID_{i}$, password $PW_{i}$, and biometrics Bio. This computer calculates $\sigma_{i}=Rep\left(Bio,\tau_{i}\right)$, $N=PW_{i}⊕h\left(ID_{i}\|\sigma_{i}\right)$, and $M^{{}^{\prime }}=h\left(N\|r_{1}\right)⊕HID_{i}$, where $r_{1}=\Omega_{i}⊕N$ and $HID_{i}=h\left(ID_{i}\|r_{1}\right)$. Subsequently, it determines whether $M^{{}^{\prime }}$ is equal to *M* stored in the smart card. If $M^{{}^{\prime }}=M$, the computer generates $r_{u}$ and timestamp $T_{1}$ and calculates $HPW_{i}=h\left(PW_{i}\|\sigma_{i}\right)$, $B_{1}=D_{3}⊕N⊕HPW_{i}$, and $B_{2}=B_{1}⊕r_{u}$. $U_{i}$ calculates $X_{UG}=h(T_{1}\|r_{u}\|HID_{i}\left\|B_{2}\right)$ and then sends $\left\{HID_{i},B_{2},X_{UG},T_{1}\right\}$ to GWN.
(2) GWN first verifies the freshness of $T_{1}$ and retrieves the corresponding $D_{1}$ from its own database according to $HID_{i}$. Thereafter, GWN calculates $B_{1}=h\left(D_{1}\|G_{j}\right)$, $r_{u}=B_{1}⊕B_{2}$, and $X_{UG}^{{}^{\prime }}=h(T_{1}\|r_{u}\|HID_{i}\left\|B_{2}\right)$. If $X_{UG}^{{}^{\prime }}$ and the received $X_{UG}$ are equal, GWN generates a random number $r_{g}$ and current timestamp $T_{2}$. Subsequently, GWN calculates $HSID_{j}=h\left(SID_{j}\|G_{j}\right)$, $B_{3}=r_{u}⊕h\left(HSID_{j}\|G_{j}\right)$, $B_{4}=D_{1}⊕h(B_{3}\|SID_{j}\left\|r_{u}\right)$, $B_{5}=r_{g}⊕h\left(D_{1}\|r_{u}\right)$, $B_{6}=B_{3}⊕PID_{j}$, and $X_{GS}=h(T_{2}\|r_{u}\|r_{g}\|SID_{j}\left\|B_{5}\right)$. Thereafter, GWN transmits $\{B_{4}$, $B_{5}$, $B_{6}$, $X_{GS}$, $T_{2}\}$ to $SN_{j}$.
(3) $SN_{j}$ verifies the freshness of $T_{2}$ and then obtains $PID_{j}$ by decrypting *L* with his or her private key pus. Thereafter, $SN_{j}$ calculates $B_{3}=B_{6}⊕PID_{j}$, $r_{u}=B_{3}⊕SG⊕PID_{j}$, $D_{1}=B_{4}⊕h(B_{3}\|SID_{j}\left\|r_{u}\right)$, $r_{g}=B_{5}⊕h\left(D_{1}\|r_{u}\right)$, and $X_{GS}^{{}^{\prime }}=h(T_{2}\|r_{u}\|r_{g}\|SID_{j}\left\|B_{5}\right)$. $SN_{J}$ determines whether $X_{GS}^{{}^{\prime }}$ is the same as the received $X_{GS}$. If so, $SN_{j}$ generates $T_{3}$, $r_{3}$, and computes $B_{7}=r_{s}⊕h(SG\|D_{1}\left\|r_{g}\right)$, $B_{8}=PID_{j}⊕B_{7}$, $X_{SG}=h(T_{3}\|r_{g}\|r_{s}\|B_{7}\left\|SG\right)$, and $X_{SU}=h(r_{u}\|r_{s}\|SID_{j}\left\|D_{1}\right)$. Finally, $SN_{j}$ calculates the session key SK as $h(r_{u}\|r_{g}\|r_{s})$. At this point, $SN_{j}$ transmits $\{B_{8}$, $X_{SG}$, $X_{SU}$, $T_{3}\}$ to GWN.
(4) GWN first verifies the freshness of $T_{3}$, and calculates $B_{7}=B_{8}⊕PID_{j}$, $SG=h\left(HSID_{j}\|G_{j}\right)⊕PID_{j}$, and $r_{s}=B_{7}⊕h(SG\|D_{1}\left\|r_{g}\right)$. Subsequently, GWN verifies the legitimacy of $SN_{j}$ by determining whether $h(T_{3}\|r_{g}\|r_{s}\|B_{7}\|SG)$ is equal to $X_{SG}$. If they are equal, GWN generates a timestamp $T_{4}$, computes $B_{9}=D_{1}⊕B_{1}$, $B_{10}=B_{9}⊕h\left(HID_{i}\|G_{j}\right)⊕r_{s}$, and $B_{11}=SID_{j}⊕h\left(B_{1}\|r_{s}\right)$, and produces a session key $SK=h(r_{u}\|r_{g}\|r_{s})$. GWN provides $X_{GU}=h(T_{4}\|r_{u}\|r_{g}\left\|B_{10}\right)$ for mutual authentications with the user and sends $\{B_{5},B_{10}$, $B_{11}$, $X_{GU}$, $X_{SU}$, $T_{4}\}$ to $U_{i}$.
(5) The computer of $U_{i}$ inspects the timestamp from GWN, and computes $r_{s}=B_{1}⊕B_{10}⊕D_{4}$ and $r_{g}=B_{5}⊕h\left(D_{1}\|r_{u}\right)$. Thereafter, it calculates $X_{GU}^{{}^{\prime }}$ and verifies whether $X_{GU}^{{}^{\prime }}=X_{GU}$. Subsequently, it calculates $X_{SU}^{{}^{\prime }}=h(r_{u}\|r_{s}\|SID_{j}\left\|D_{1}\right)$, where $SID_{j}=B_{11}⊕h\left(B_{1}\|r_{s}\right)$. At this time, $U_{i}$ can successfully calculate the session key $SK=h(r_{u}\|r_{g}\|r_{s})$. Obviously, $U_{i}$, GWN, and $SN_{j}$ have the same session key at this point.

## 4. Security Analysis

This section first describes the capabilities that the attacker A may possess. Subsequently, we demonstrate that our method is secure against different types of attacks. Finally, we use the Real-or-Random (ROR) model to show that our LAP-IoHT protocol is provably secure.

### 4.1. Adversary Model

We consider the well-known Dolev–Yao (DY) adversary model [45] and assume that an attacker A has the following capabilities:

(1) A can eavesdrop, block, replay, alter, and delete messages that are sent over a public channel.
(2) A can steal the smart card or smart device of a user and obtain the information stored therein.
(3) A can capture a sensor node to extract the information stored therein.
(4) A can obtain the long-term key of the gateway and acquire the contents stored therein as an internal privileged person.

### 4.2. Protection against Well-Known Attacks

#### 4.2.1. Replay Attack

In LAP-IoHT, messages that are transmitted via a public channel have timestamps, such as $T_{1}$, $T_{2}$, $T_{3}$, and $T_{4}$. These timestamps ensure the freshness of the messages and resist replay attacks. Moreover, $X_{UG}$, $X_{GS}$, $X_{SG}$, $X_{SU}$, and $X_{GU}$ include random numbers. Timestamps and random numbers are two effective means of preventing replay attacks. Thus, LAP-IoHT is resistant against replay attacks.

#### 4.2.2. User Impersonation Attack

Assume that A can obtain the private key $G_{j}$ of GWN. Even if A intercepts the parameters $T_{1}$, $HID_{i}$, and $B_{2}$ via a public channel, A still cannot obtain $r_{u}$ because A cannot obtain $B_{1}$ and $D_{1}$. Therefore, A fails to calculate $X_{UG}$, cannot pass the authentication of GWN, and cannot imitate $U_{i}$ for communication. Thus, LAP-IoHT can effectively resist user impersonation attacks.

#### 4.2.3. Server Impersonation Attack

Suppose that A can obtain a smart card for $U_{i}$. However, A does not know the value of $SID_{j}$ and the private key $G_{j}$ of the gateway; therefore, A cannot pass the authentication of $SN_{j}$ by computing $X_{GS}$ and cannot successfully imitate the gateway. Hence, our protocol can defend against server impersonation attacks.

#### 4.2.4. Privileged Insider Attack

If A is an insider of GWN, A can obtain $HID_{i}$, $D_{1}$, $SID_{j}$, and $PID_{j}$, which are stored in the database of GWN. However, A cannot successfully obtain the session key because he or she does not know $r_{u}$, $r_{g}$, and $r_{s}$. Thus, the proposed protocol can defend against privileged insider attacks. Therefore, we can state that the proposed protocol is secure against insider attacks.

#### 4.2.5. Known Session Specific Temporary Information Attack

We assume that the temporary random number $r_{u}$ is obtained using A. If A wishes to calculate the session key SK, three parameters $r_{u},r_{g},$ and $r_{s}$ are required. However, A cannot know $r_{g}$ because he or she cannot obtain $PID_{j}$. Furthermore, A cannot obtain $r_{s}$. Thus, our protocol is not affected by temporary information leakage.

#### 4.2.6. Stolen Smart Card Attack

A obtains $\left\{D_{1},D_{3},D_{4},\Omega_{i},M\right\}$ stored in the smart card that he or she has stolen. Even if A knows $B_{2}$ and $D_{1}$, A cannot obtain $B_{1}$ because he or she cannot obtain $G_{j}$. This implies that A cannot pass the server verification let alone establish a communication session key with GWN. Thus, LAP-IoHT is resistant against smart card theft attacks.

#### 4.2.7. Perfect Forward Security

If A knows the $G_{j}$ of the gateway when calculating the random number $r_{u}=B_{1}⊕B_{2}$, $B_{2}$ can intercept the transmitted information and the other parameter $B_{1}=h\left(D_{1}\|G_{j}\right)$. $G_{j}$ is already known by A, but as $D_{1}=h\left(HID_{i}\|N\right)$, A cannot obtain *N* and $HID_{i}$ and, hence, cannot know $D_{1}$. Since A cannot calculate $r_{u}$, he or she cannot obtain session key SK. Therefore, our protocol provides perfect forward security.

### 4.3. ROR Security Analysis

The ROR (Real-or-Random) model is a widely used security-proof method. The ROR model can obtain the probability of successfully breaking session key SK through several different game rounds. Therefore, we use the ROR model to perform a formal security analysis to demonstrate the security and accuracy of the protocol.

#### 4.3.1. ROR Model

Our protocol comprises three entities: $U_{i}$, GWN, and $S_{j}$. We use $\Pi_{U_{i}}^{x}$, $\Pi_{GWN}^{y}$, and $\Pi_{S_{j}}^{z}$ to denote the x-th user, y-th gateway, and z-th sensor nodes, respectively, such that $R=\{\Pi_{U_{i}}^{x}$, $\Pi_{GWN}^{y}$, and $\Pi_{S_{j}}^{z}\}$. Suppose that attacker A can execute the following queries:

Execute(R): When this query is executed, A can intercept the messages that are transmitted among entities $U_{i}$, GWN, and $S_{j}$ over the public channel.

Send(R,M): By executing this query, A can send message *M* to *R* and receive the response message from *R*.

Hash(String): Through this operation, A can obtain the hash value of a fixed-length string after inputting it.

Corrupt(R): By executing this query, A obtains the private value of an entity, such as long-term key, generated temporary information, or parameters that are stored in a smart card.

Test(R): Assume that A executes this query and can determine the security of the session key by tossing coin C. If C = 1, A obtains the correct session key. Otherwise, A receives a random string.

Theorem 1: In the ROR model, we use $Adv_{A}^{P}$ as a function of the attacker’s ability to compromise the protocol through query operations; that is, the probability that A can obtain the session key $Adv_{A}^{P}\leq q_{h}^{2}/\left|H\right|+q_{s}/2^{t-1}\left|D\right|$, where $q_{h}$ and $q_{s}$ represent the number of times to perform the Hash and Send queries, respectively, |H| and |D| represent the space range and dictionary size corresponding to the hash operation, respectively, and *t* represents the number of bits of biological information in the protocol.

#### 4.3.2. Security Proof

To prove the accuracy of Theorem 1, we performed four rounds of game $GM_{i}\left(i=0,1,2,3\right)$, where $Succ_{A}^{GM_{i}}$ denotes the probability of the attacker A winning in each round of the game. The details of the game are as follows.

$GM_{0}$: At the beginning of the game, A only needs to determine bit *b* and does not perform any query operation. Therefore, we can obtain

(1) $$Adv_{A}^{P}=\left|2Pr\left[Succ_{A}^{GM_{0}}\right]-1\right|.$$

$GM_{1}$: $GM_{1}$ performs a wiretap operation on top of $GM_{0}$. In this round, A can only steal messages that are transmitted on the common channels $\left\{HID_{i},B_{2},X_{UG},T_{1}\right\}$, $\left\{B_{4},B_{5},B_{6},X_{GS},T_{2}\right\}$, $\left\{B_{8},X_{SG},X_{SU},T_{3}\right\}$, and $\left\{B_{5},B_{10},B_{11},X_{GU},X_{SU},T_{4}\right\}$. A cannot execute the Test queries to obtain the session key $SK=h(r_{u}\|r_{g}\|r_{s})$ during communication because the values of the random numbers $r_{u}$, $r_{g}$, and $r_{s}$ cannot be obtained based only on the information in the common channels. Therefore, the probability of A winning the game after performing an Execute query is equal to $GM_{0}$.

(2) $$Pr\left[Succ_{A}^{GM_{1}}\right]=Pr\left[Succ_{A}^{GM_{0}}\right].$$

$GM_{2}$: $GM_{2}$ is the third round of the game, in which the Hash query and Send operation have already occurred in $GM_{1}$. During the game, forgery is not possible because $B_{4}$, $X_{UG}$, $B_{4}$, $B_{5}$, $X_{GS}$, $B_{1}1$, $X_{SG}$, $X_{SU}$, and $X_{GU}$ are encrypted using hash functions. Moreover, the important parameters $r_{u}$, $r_{g}$, and $r_{s}$, which constitute the session key, are random in all sessions and do not cause hash conflicts. Thus, according to the birthday paradox, we obtain

(3) $$|Pr\left[Succ_{A}^{GM_{2}}\right]-Pr\left[Succ_{A}^{GM_{1}}\right]|\leq q_{h}^{2}/2\left|H\right|.$$

$GM_{3}$: In this round, the Corrupt query is executed and the attacker A can obtain the private value of an entity, such as {SG,L}, $\left\{D_{1},D_{3},D_{4},\Omega_{i},M\right\}$, or $\left\{SID_{j},PID_{j},HID_{i},D_{1}\right\}$. Moreover, A attempts to guess $ID_{i}$ and $PW_{i}$; however, even if A can successfully guess $ID_{i}$ and $PW_{i}$ simultaneously, he or she still cannot obtain the random number $r_{u}$. Since $r_{u}=B_{1}⊕B_{2}$, $B_{1}=D_{3}⊕N⊕HPW_{i}$, $N=PW_{i}⊕h\left(ID_{i}\|\sigma_{i}\right)$, $\sigma_{i}=Rep\left(Bio,\tau_{i}\right)$, and the probability of the biometric being estimated is $1/2^{t}$, A cannot obtain the biological eigenvalue Bio. If A can only enter the code a finite number of times, we know that

(4) $$|Pr\left[Succ_{A}^{GM_{3}}\right]-Pr\left[Succ_{A}^{GM_{2}}\right]|\leq q_{s}/2^{t}\left|D\right|.$$

Since A can only win the game if the correct bit *b* is guessed, we obtain

(5) $$|Pr[Succ_{A}^{GM_{3}}]|=1/2.$$

Using Equations (1)–(5) above, we obtain

(6) $$\begin{array}{c} 1/2Adv_{A}^{P}=\left|Pr\left[Succ_{A}^{GM_{0}}\right]-1/2\right|\\ =|Pr\left[Succ_{A}^{GM_{1}}\right]-Pr\left[Succ_{A}^{GM_{3}}\right]|\\ \leq |Pr\left[Succ_{A}^{GM_{2}}\right]-Pr\left[Succ_{A}^{GM_{1}}\right]|+|Pr\left[Succ_{A}^{GM_{3}}\right]-Pr\left[Succ_{A}^{GM_{2}}\right]|\\ =q_{h}^{2}/2|H|+q_{s}/2^{t}\left|D\right|. \end{array}$$

Ultimately, we can obtain $Adv_{A}^{P}\leq q_{h}^{2}/\left|H\right|+q_{s}/2^{t-1}\left|D\right|$.

### 4.4. Security Comparisons

We compare LAP-IoHT with other related protocols with similar architectures, such as those of Kumar et al. [43], Yu et al. [44], Amin et al. [36], Challa et al. [37], Aghili et al. [39], and Preeti et al. [38]. We set the following representations: A1: resist replay attack; A2: resist impersonation attack; A3: resist privileged insider attack; A4: perfect forward security; A5: resist known session specific temporary information attack; A6: resist stolen smart card attack; A7: resist offline password guessing attack; A8: resist sensor node capture attack; A9: resist de-synchronization attack; A10: resist session key disclosure attack. “Y” indicates that the protocol is invulnerable to this attack, and “N” indicates that the protocol is vulnerable to this attack. The results in Table 3 demonstrate that, with the continual development of technology and various attack methods, the other related protocols will be affected by the above attacks. Compared to these protocols, our method exhibits better security and sufficient advantages in resisting the above attacks to guarantee the security of communication sessions.

## 5. Performance Comparison

In this section, we evaluate the performance of the proposed LAP-IoHT protocol by performing comparisons with other protocols, such as those proposed by Kumar et al. [43], Yu et al. [44], Amin et al. [36], Challa et al. [37], Aghili et al. [39], and Preeti et al. [38], in terms of the computation time and communication cost.

We used different devices to obtain the computation time and communication cost required for the certification stage in the performance comparison. We used a mobile phone, laptop computer, and desktop computer to simulate the user, gateway, and sensor nodes, respectively. The relevant parameters for the three devices are listed in Table 4. Table 5 presents the times required by different devices to perform certain operations. $T_{H}$ denotes the time required to perform a single hash function operation, $T_{SED}$ denotes the time required to perform a single symmetric encryption or decryption operation, $T_{FE}$ denotes the time required to perform a single fuzzy extraction operation, $T_{ASED}$ denotes the time required to perform a single asymmetric encryption or decryption operation, $T_{S}$ denotes the time required to execute the digital signature operation, and $T_{PM}$ denotes the time required to perform an elliptic curve point multiplication operation. As the communication times required by the connection and XOR operations are insignificant compared to the other operations, these can be ignored. Table 6 presents a comparison of the communication times of our proposed protocol and other similar protocols. Several communication costs arise in the communication process, and asymmetric encryption or decryption has an enormous overhead of 1024 bits. The length required for the elliptic curve point multiplication operation is 320 bits; the length of each block for symmetric encryption or decryption is 256 bits; the hash values and random numbers all have similar lengths of 160 bits; the identity, password, and biometrics are all 128 bits in length; the timestamps require a length of 32 bits. In Table 7, we compare the communication overheads of multiple protocols to determine the specific communication cost.

### 5.1. Computation Time

We use three devices to determine the computation time and communication cost. The times required to perform elliptic curve point multiplication, symmetric encryption/decryption, asymmetric encryption/decryption, single fuzzy extraction, and hash functions vary on different devices. Furthermore, the computation times required for the connection and XOR operations are insignificant compared to the other operations; thus, we ignore these in our evaluation.

The computation times of the proposed protocol and other similar protocols are listed in Table 6. Table 6 shows the computation costs of all protocols. The most time-consuming protocol is the protocol proposed by Kumar et al. [43], which includes elliptic curve point multiplication and digital signature operations. The protocol proposed by Yu et al. [44] is the least time consuming. Although our proposed protocol includes fuzzy extraction and asymmetric operations in the login and authentication processes, its computation time is relatively short.

### 5.2. Communication Cost

We assume that the output of asymmetric encryption/decryption is 1024 bits; the length required for the elliptic curve point multiplication operation is 320 bits; each block for symmetric encryption/decryption is 256 bits; the hashed value and random number are 160 bits; the identity, password, and biometrics are all 128 bits in length; the timestamps require a length of 32 bits.

According to Table 7, we can determine the communication costs of all the protocols. The communication costs of the protocols proposed by Kumar et al. [43], Yu et al. [44], Amin et al. [36], Challa et al. [37], Aghili et al. [39], and Preeti et al. [38] are 6720 bits (256 ∗ 7 + 32 + 256 ∗ 6 + 32 + 256 ∗ 7 + 32 + 32 + 256 ∗ 5 + 160 + 32), 2560 bits (160 + 160 + 160 + 160 + 32 + 160 + 160 + 32 + 160 + 160 + 160 + 32 + 160 + 160 + 32 + 160 + 160 + 160 + 160 + 32, 3040 bits (128 + 320 + 160 + 160 + 32 + 160 + 256 ∗ 3 + 320 + 32 + 256hl∗3 + 32 + 160), 1408 bits (160 + 160 + 320 + 160 + 32 + 160 + 32 + 32 + 160 + 160 + 32), 2272 bits (160 + 160 + 160 + 320 + 32 + 160 + 160 + 32 + 32 + 160 + 160 + 320 + 32 + 32 + 160 + 160 + 32), and 2016 bits (160 + 160 + 160 + 160 + 128 + 32 + 160 + 160 + 160 + 32 + 160 + 160 + 32 + 160 + 160 + 32), respectively. The communication cost of our proposed protocol is 2496 bits (128 + 160 + 160 + 32 + 160 + 160 + 160 + 160 + 32 + 160 + 160 + 160 + 32 + 160 + 160 + 160 + 160 + 160 + 32).

**Figure 5:**
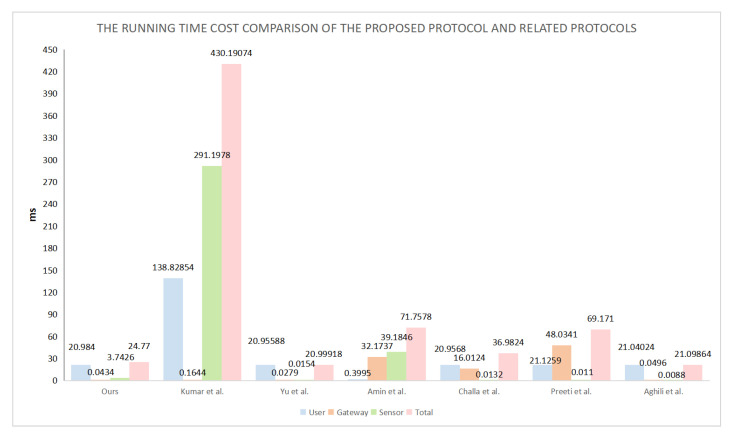
Running times.

**Figure 6:**
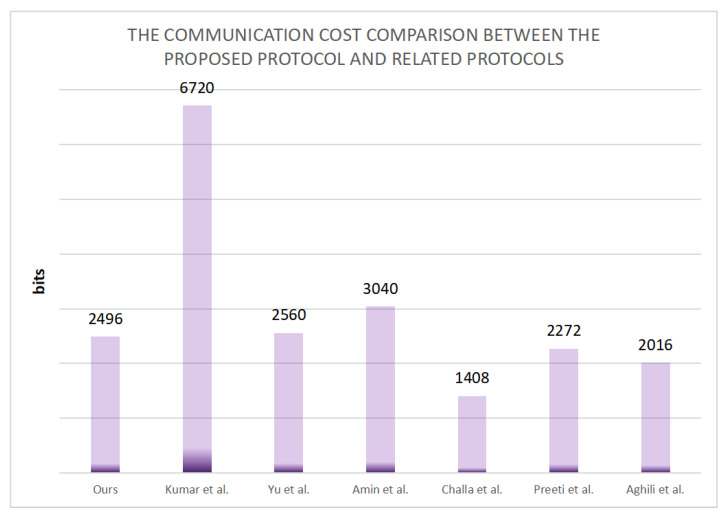
Communication costs.

Figure 5 and Figure 6 compare the LAP-IoHT protocol with the other related protocols in terms of the computation times and communication costs. Although the communication costs of the LAP-IoHT protocol are higher than those of the protocols proposed by Challa et al. [37], Aghili et al. [39], and Preeti et al. [38], the run time of LAP-IoHT is much lower [37,38]. Moreover, the security of LAP-IoHT is higher than those of all three [37,38,39]. Furthermore, although the protocols proposed by Kumar et al. [43] and Yu et al. [44] are more secure, they do not offer any advantages in terms of communication costs. Therefore, it is easy to conclude that LAP-IoHT performs better than the related protocols. More importantly, it can be observed from Table 3 that LAP-IoHT has excellent security advantages. It can effectively resist various attacks, thereby providing security for communication sessions.

## 6. Conclusions

Internet of Health Things (IoHT), which promotes intelligent healthcare, plays a pivotal role in the future e-healthcare environment. Due to its high sensitivity, the health data transmitted through a public channel should be protected from unauthorized access. This means that an authentication protocol is essential. This paper presented a more secure and reliable authentication protocol called LAP-IoHT for the Internet of Health Things. LAP-IoHT provides mutual authentication among users, sensors, and a gateway over a public channel. Moreover, a user and a sensor can establish a common session key after a protocol run. By using the ROR model and performing an informal analysis, it was proven that LAP-IoHT has adequate security and reliability as well as sufficient ability to resist various attacks. Furthermore, we compared LAP-IoHT with related protocols and found that our protocol is at the mid-to-upstream level in terms of time and communication costs, exhibiting a significant performance advantage. In summary, the proposed protocol offers specific practical value in the current environment and has more robust adaptability relative to the future development of IoHT.

## Figures and Tables

**Table 1:** A summary of the application of the Internet of Things in the medical industry.

Protocols	Advantages	Limitations
Amin et al. [36]	(1) Resist impersonation attack (2) Resist smart card stolen attack (3) Resist replay attack	(1) Cannot resist privileged insider attack (2) Cannot resist offline password guessing attack
Challa et al. [37]	(1) Provide user anonymity (2) Resist offline password guessing attack (3) Resist man-in-the middle attack	(1) Cannot resist sensor node capture attack
Preeti et al. [38]	(1) Provide mutual authentication (2) Resist DoS attack (3) Resist known-session-specific temporary information attack	(1) Cannot provide perfect forward security (2) Cannot resist sensor node capture attack
Aghili et al. [39]	(1) Provide user untraceability (2) Resist de-synchronization attack (3) Resist DoS attack	(1) Cannot provide perfect forward security (2) Cannot resist malicious sensor attack (3) Cannot resist server impersonation attack
Amintoosi et al. [40]	(1) Resist known-session-specific temporary information attack (2) Provide perfect forward security (3) Resist privileged insider attack	–
Gupta et al. [41]	(1) Provide perfect forward security (2) Resist impersonation attack (3) Provide anonymity and untraceability	(1) Cannot resist privileged insider attack (2) Cannot resist offline password guessing attack (3) Cannot resist de-synchronization attack
Hajian et al. [42]	(1) Resist replay attack (2) Resist privileged insider attack (3) Resist de-synchronization attack	(1) Cannot provide perfect forward security (2) Cannot resist session key disclosure attack (3) Cannot resist impersonation attack
Kumar et al. [43]	(1) Resist privileged insider attack (2) Resist man-in-the-middle attack (3) Resist replay attack	–
Yu et al. [44]	(1) Provide user untraceability and anonymity (2) Resist session key disclosure attack (3) Provide mutual authentication	–

**Table 2:** Notation definitions.

Notations	Descriptions
$U_{i}$	*i*th user
$ID_{i}$	Identity of $U_{i}$
$PW_{i}$	Password of $U_{i}$
Bio	Biometrics of $U_{i}$
$SN_{j}$	*j*th sensor node
$SID_{j}$	Identity of $SN_{j}$
GWN	Gateway node
$G_{j}$	Private key of GWN
pbs	Public key of $SN_{j}$
pvs	Private key of $SN_{j}$
SK	Session key
$T_{s}$	Time stamp, where *s* = 1, 2, 3, 4
$r_{1},r_{u},r_{g},r_{s}$	Temporary random number
⊕	XOR operation
‖	Concatenate operation
*h*(·)	Hash function
Gen(·)/Rep(·)	Fuzzy extractor/reproduction function
ENC/DEC	Asymmetric encryption/decryption
→	The public channel
⇒	The secure channel
A	Adversary

**Table 3:** Comparisons of security.

Protocols	A1	A2	A3	A4	A5	A6	A7	A8	A9	A10
Ours	*Y*	*Y*	*Y*	*Y*	*Y*	*Y*	*Y*	*Y*	*Y*	*Y*
Kumar et al. [43]	*Y*	*Y*	*Y*	*Y*	*Y*	*Y*	*Y*	*Y*	*Y*	*Y*
Yu et al. [44]	*Y*	*Y*	*Y*	*Y*	*Y*	*Y*	*Y*	*Y*	*Y*	*Y*
Amin et al. [36]	*Y*	*Y*	*N*	*Y*	*Y*	*Y*	*N*	*Y*	*Y*	*Y*
Challa et al. [37]	*Y*	*Y*	*Y*	*Y*	*Y*	*Y*	*Y*	*N*	*Y*	*Y*
Preeti et al. [38]	*Y*	*Y*	*Y*	*N*	*Y*	*Y*	*Y*	*N*	*Y*	*Y*
Aghili et al. [39]	*Y*	*N*	*N*	*Y*	*Y*	*Y*	*Y*	*Y*	*Y*	*Y*

**Table 4:** Parameters of the devices.

Devices	Model	Operating System	Memory	Processor
mobile phone	MI 8	Android	6 GB	Qualcomm Snapdragon 845
laptop computer	DELL G15 5510	Windows 10	16 GB	Intel(R) Core(TM)i7-10870H
desktop computer	LENOVO 90M2A0A6CD	Windows 10	8 GB	Intel(R) Core(TM)i5-9500

**Table 5:** Execution time of operations.

Operations	MI 8	DELL G15 5510	LENOVO 90M2A0A6CD
$T_{FE}$	20.7028 ms	2.2823 ms	1.6197 ms
$T_{ASED}$	47.6405 ms	5.2520 ms	3.7272 ms
$T_{PM}$	0.00044 ms	16 ms	13 ms
$T_{SED}$	0.2009 ms	0.1551 ms	0.0879 ms
$T_{H}$	0.02812 ms	0.0031 ms	0.0022 ms
$T_{S}$	69 ms	270 ms	139 ms

**Table 6:** Comparison of time.

Protocols	User	Gateway	SensorNode	Total Computation
				(ms)
Ours	$T_{FE}+10T_{H}$	$14T_{H}$	$T_{ASED}+7T_{H}$	24.77
Kumar et al. [43]	$2T_{PM}+8T_{H}+2T_{S}+3T_{SED}$	$T_{SED}+3T_{H}$	$T_{PM}+10T_{H}+2T_{S}+2T_{SED}$	370.19074
Yu et al. [44]	$T_{FE}+9T_{H}$	$9T_{H}$	$7T_{H}$	20.99918
Amin et al. [36]	$T_{SED}+4T_{PM}+7T_{H}$	$T_{SED}+2T_{PM}+6T_{H}$	$2T_{SED}+3T_{PM}+4T_{H}$	71.7578
Challa et al. [37]	$T_{FE}+2T_{PM}+9T_{H}$	$T_{PM}+4T_{H}$	$6T_{H}$	36.9824
Preeti et al. [38]	$T_{FE}+3T_{PM}+15T_{H}$	$3T_{PM}+11T_{H}$	$5T_{H}$	69.171
Aghili et al. [39]	$T_{FE}+12T_{H}$	$16T_{H}$	$4T_{H}$	21.09864

**Table 7:** Comparison of cost.

Protocols	User	Gateway	SensorNode	Total Communication Cost (bits)	Number of Messages
Ours	480	1504	512	2496	4
Kumar et al. [43]	1824	3424	1472	6720	4
Yu et al. [44]	672	1216	672	2560	5
Amin et al. [36]	960	1280	800	3040	4
Challa et al. [37]	832	224	352	1408	3
Preeti et al. [38]	832	1088	352	2272	4
Aghili et al. [39]	800	864	4352	2016	4

## Data Availability

Not applicable.

## Abbreviations

The following abbreviations are used in this manuscript:

IoT	Internet of Things;
WSN	Wireless sensor network;
IoHT	Internet of Health Things;
ECG	Electrocardiogram;
EMG	Electromyography;
EEG	Electroencephalogram;
DY	Dolev–Yao;
ROR	Real-or-Random;
XOR	Exclusive OR;
DoS	Denial of service.
